# Disproportionation of H_2_O_2_ Mediated by Diiron-Peroxo Complexes as Catalase Mimics

**DOI:** 10.3390/molecules26154501

**Published:** 2021-07-26

**Authors:** Dóra Lakk-Bogáth, Patrik Török, Flóra Viktória Csendes, Soma Keszei, Beatrix Gantner, József Kaizer

**Affiliations:** Research Group of Bioorganic and Biocoordination Chemistry, University of Pannonia, H-8201 Veszprém, Hungary; lakkd@almos.uni-pannon.hu (D.L.-B.); patriktrk6@gmail.com (P.T.); csvflora@gmail.com (F.V.C.); kornflakes09@gmail.com (S.K.); gantnerbea@gmail.com (B.G.)

**Keywords:** catalase mimics, peroxide, diiron-peroxo complexes, structure/activity, kinetic studies

## Abstract

Heme iron and nonheme dimanganese catalases protect biological systems against oxidative damage caused by hydrogen peroxide. Rubrerythrins are ferritine-like nonheme diiron proteins, which are structurally and mechanistically distinct from the heme-type catalase but similar to a dimanganese KatB enzyme. In order to gain more insight into the mechanism of this curious enzyme reaction, non-heme structural and functional models were carried out by the use of mononuclear [Fe^II^(L_1–4_)(solvent)_3_](ClO_4_)_2_ (**1**–**4**) (L_1_ = 1,3-bis(2-pyridyl-imino)isoindoline, L_2_ = 1,3-bis(4′-methyl-2-pyridyl-imino)isoindoline, L_3_ = 1,3-bis(4′-Chloro-2-pyridyl-imino)isoindoline, L_4_ = 1,3-bis(5′-chloro-2-pyridyl-imino)isoindoline) complexes as catalysts, where the possible reactive intermediates, diiron-perroxo [Fe^III^_2_(μ-O)(μ-1,2-O_2_)(L_1_-L_4_)_2_(Solv)_2_]^2+^ (**5**–**8**) complexes are known and well-characterized. All the complexes displayed catalase-like activity, which provided clear evidence for the formation of diiron-peroxo species during the catalytic cycle. We also found that the fine-tuning of iron redox states is a critical issue, both the formation rate and the reactivity of the diiron-peroxo species showed linear correlation with the Fe^III^/Fe^II^ redox potentials. Their stability and reactivity towards H_2_O_2_ was also investigated and based on kinetic and mechanistic studies a plausible mechanism, including a rate-determining hydrogen atom transfer between the H_2_O_2_ and diiron-peroxo species, was proposed. The present results provide one of the first examples of a nonheme diiron-peroxo complex, which shows a catalase-like reaction.

## 1. Introduction

Nonheme manganese catalases (MnCAT), as alternatives to the heme-containing catalases, contain a binuclear active sites and deplete hydrogen peroxide in cells through a ping-pong mechanism interconventing between Mn_2_(II,II) and Mn_2_(III,III) states in a two-electron catalytic cycle [1,2]. Manganese catalase enzymes such as *Lactobacillus plantarum* (LPC) [3,4], *Thermus thermophilus* (TTC) [5,6], *Thermoleophilium album* (TAC) [7], and *Pyrobaculum calidifontis VA1* (PCC) [8] have been isolated and characterized from both bacteria and archae. Despite their large numbers and importance, few catalase enzyme structures are known and they can also be sub-divided into two groups, each with a different active site.

**Scheme 1:**
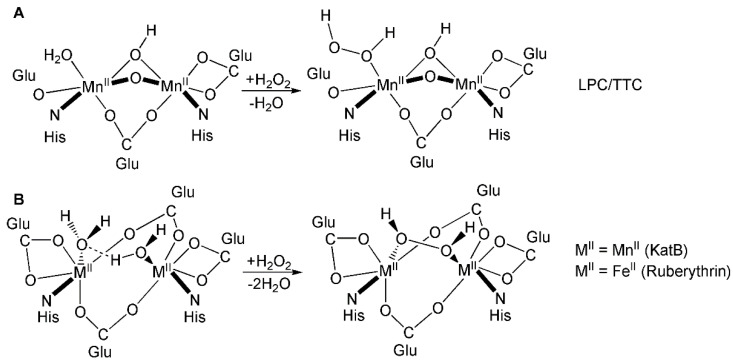
Binding mode of H_2_O_2_ in nonheme dimanganese and diiron enzymes.

Among them both LPC and TTC have a dimanganese catalytic core with Glu_3_His_2_ ligands coupled by two single-atom solvent bridges (Scheme 1A) [4,9]. In contrast to the above structure, the cyanobacterial manganese catalase (KatB) from *Anabaena* shows a markedly different active site with canonical Glu_4_His_2_ coordination geometry and two terminal water molecules (Scheme 1B). However, it closely resembles the active site of the ferritin-like ruberythrin (RBR), which is a nonheme diiron protein [10]. The Mn-Mn distances (~3.8 Å) in KatB are similar to that was observed for the iron-containing ribonucleotide reductase R2 (3.6–3.7 Å), but much shorter distances have been found for LPC and TTC (3.0–3.1 Å). The H_2_O_2_ binds terminally to one of the Mn center in LPC/TTC, while in KatB and RBR, the symmetrical active sites and the relatively long M-M distances favor its μ-1,2 binding mode [11]. These enzymes, coupled with superoxide dimutates (SOD), are responsible for the full detoxification of O_2_^•─^ through the formation of H_2_O_2_ and its redox disproportionation into water and dioxygen. H_2_O_2_ can also be classified as a ROS particle, since it readily reacts with reduced transition metals and results in highly reactive HO^•^ radical via Fenton-like reactions. Many rationally designed synthetic biomimetics are being developed against oxidative stress to treat various diseases, including Alzheimer’s, cancer, aging, neurodegenerative, inflammatory and heart diseases [12,13,14,15,16,17,18,19,20,21]. The identification and study of the structure of the above enzymes can greatly contribute to the design of new catalase mimetics for potential therapeutic use. The structural and functional investigations on low molecular-weight copper, manganese and iron containing model compounds that are focused mainly on the effect of the used metals and their ligand environment (donor sites) on redox potential and through it on reactivity. Based on the literature data, various Schiff base, aminopyridine and azamacrocyclic derivatives have proven to be the most popular and practical ligands [12,13,14,15,16,17,18,19,20,21]. Considering the reactivity of manganese catalases (KatB) toward H_2_O_2_, including a ping-pong mechanism interconventing between Mn_2_(II,II) and Mn_2_(III,III) states in a two-electron catalytic cycle, a similar interaction with H_2_O_2_ has not been reported for diiron(III) peroxo intermediates which may be proposed in the catalytic cycles of RBR. Progress in recent years has made a number of diiron(III)-peroxo complexes available that can be generated by stoichiometric amount of H_2_O_2_ [22,23,24,25,26,27]. Therefore, the opportunity has arisen to investigate both electrophilic and nucleophilic reactivity towards various substrates, such as phenols, *N*,*N*-dimethylanilines, and various aldehydes, respectively [25,26]. The differences in reactivity observed in the investigated systems, as well as the stability of the peroxo intermediates can in principle be explained by the disproportionation of H_2_O_2_ as a competing process. Clarification of the relationship between catalase-like activity and catalytic oxidation is an important factor in the design and development of high efficiency catalytic systems. In addition, the investigation of the metal-based disproportionation of H_2_O_2_ may help us to understand the nature of the catalytically active species present both, in the enzymatic and biomimetic systems. We have reported that the [Fe^II^(L_1_)(solvent)_3_](ClO_4_)_2_ (**1**) is an efficient catalyst for the oxidation of thioanisoles and benzyl alcohols by the use of H_2_O_2_ as cooxidant, where a metastabile green species (**5**) with a Fe^III^(μ-O)(μ-1,2-O_2_)Fe^III^ core was observed and characterized by UV-Vis, EPR, rRaman, and X-ray absorption spectroscopic measurements [24]. The reactivity of **5** was also published both in nucleophilic (deformylation of aldehydes) and electrophilic (oxidation of phenols) reactions [27].

**Scheme 2:**
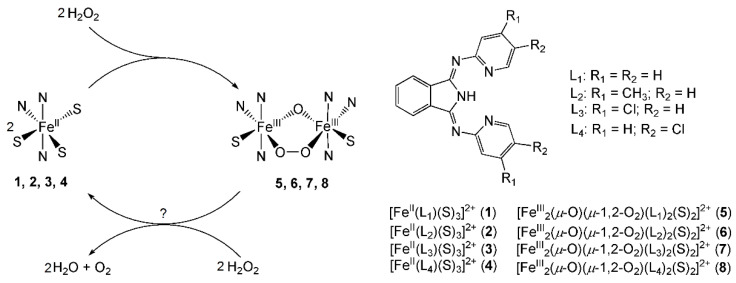
Structural formulae for the ligands and their complexes used in the disproportionation reaction of H_2_O_2_.

As a possible functional model of RBR, we recently investigated the reactivity of the peroxo adduct [Fe_2_(μ-O_2_)(MeBzim-Py)_4_(CH_3_CN)]^4+^ (MeBzim-Py = (2-(2′-pyridyl)-*N*-methylbenzimidazole) with H_2_O_2_ and found direct kinetic and computational evidence for the formation of low-spin oxoiron(IV) via dissociation process [22,23]. As a continuation of this tudy, the previously reported and fully characterized Fe^III^_2_(μ-O)(μ-1,2-O_2_)(IndH)_2_(Solv)_2_]^2+^ (IndH = L_1_ = 1,3-bis(2-pyridyl-imino)-isoindolines) [24,25] and their substituted isoindolin-containing derivatives (L_2_–L_4_, **2**–**4**) were chosen as model compounds, in which the dissociation of the diiron(III) core can be ruled out due to the μ-oxo-bridge, and investigated its reactivity towards H_2_O_2_ to gain insight into the mechanism of RBR enzymes (Scheme 2).

## 2. Results and Discussions

**Figure 1:**
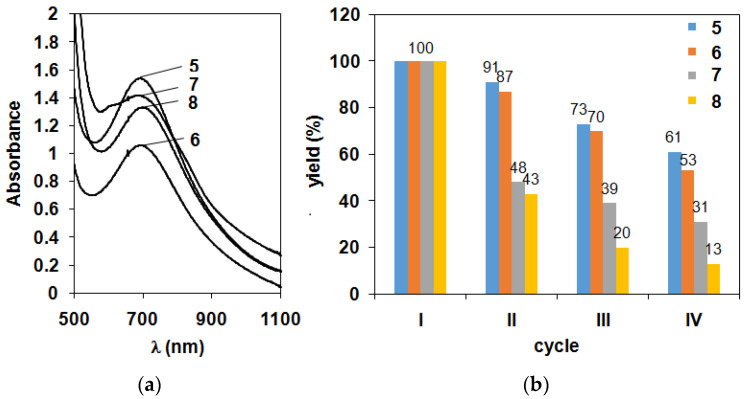
(**a**) Characteristic UV-Vis spectra of Fe^III^_2_(μ-O)(μ-1,2-O_2_)(L_1–4_)_2_(Solv)_2_]^2+^ (**5**–**8**) intermediates in CH_3_CN. (**b**) Dependence of the yield of peroxo complexes (**5**–**8**) on the number of cycles. [Fe]_0_ = 2 mM, [H_2_O_2_]_0_ = 24 mM (4x) in CH_3_CN at 20 °C.

**Figure 2:**
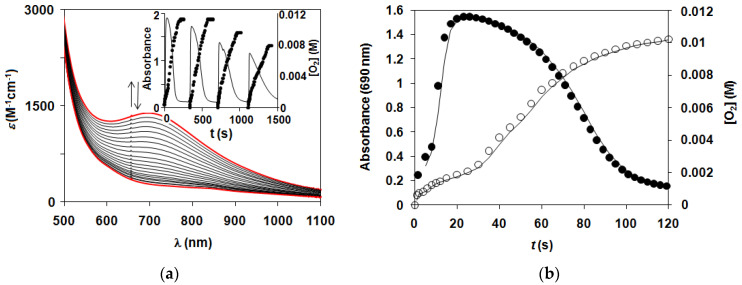
(**a**) UV-Vis spectral changes of **5** during the **1**-catalyzed disproportionation of H_2_O_2_. The inset shows the the time course of the formation and decay of **5** and the parallel formation of dioxygen for 5 cycles. [**5**]_0_ = 2 mM, [H_2_O_2_]_0_ = 24 mM (4×) in CH_3_CN at 20 °C (**b**) Time course for the H_2_O_2_ (24 mM) oxidation by **5** (2 mM), monitored at 690 nm in CH_3_CN at 20 °C and by volumetrically determined amounts of evolved dioxygen.

In view of the above results, further studies were planned to elucidate the mechanism of both the diiron(III)-peroxo formation and its decay mediated by H_2_O_2_. Complex **5** from its precursor complex **1** can be generated even at room temperature by the use of excess of H_2_O_2_ in CH_3_CN. Its formation and decay process was followed as an increase and decrease in absorbance at 690 nm (*ε* = 1500 M^−1^ cm^−1^ per Fe ion and assuming 100% conversion), which can be assigned as a charge transfer between Fe^III^ and the O_2_^2−^ ligand (Figure 1a) [24]. Similar spectral feature can be observed for the complexes **6**–**8**: 691 nm (*ε* = 1404 M^−1^ cm^−1^), 692 nm (*ε* = 1460 M^−1^ cm^−1^), and 693 nm (*ε* = 1308 M^−1^ cm^−1^), respectively. It is worth noting that the green species (**5**), after decomposition, can be regenerated 2–3 times by adding additional H_2_O_2_ without significant decrease on the yields (Table 1, Figure 1b and inset in Figure 2a). Similar behavior and yields were observed for **6**, but much lower values were obtained for **7** and **8**. These results are consistent with the stability and/or the reactivity of the intermediates at high concentrations of added water, providing further information for the possible role of the binding water during the formation of the reactive oxidant.

Preliminary kinetic studies were performed at 20 °C to establish the role of the observed diiron(III)-peroxo intermediates in the catalytic disproportionation reaction of H_2_O_2_. The formation and decomposition of **5** in the presence of H_2_O_2_ was monitored by following the rise and fall of its visible chromophore (Table 2 and Table 3), as well as the appearance of the dioxygen formed by gas volumetric methods (Figure 2b). Dioxygen formation started after a short lag phase during which the peroxo-adduct (**5**) accumulated. The lag phase was followed by a linear dioxygen formation period during which time **5** persisted in a pseudo-steady-state. This profile is similar to that found for the water-assisted decay of Fe^III^(H_2_O)(OOH) intermediate into the reactive Fe^V^(O)(OH) species [28]. Upon depletion of H_2_O_2_, **5** decomposed rapidly and the dioxygen formation ceased. The yield of dioxygen, TON (turnover number = mol H_2_O_2_/mol catalyst) and the TOF (turnover frequency = mol H_2_O_2_/mol catalyst/h) values were determined to be Yield = 85%, TON = 10.4 and TOF = 288 h^−1^ as a result of volumetric method via the measurements of dioxygen evolution from the reaction mixture ([H_2_O_2_] = 0.243 M and [1] = 0.002 M) at 20 °C in CH_3_CN. Similar values were obtained for the complexes **2**, **3** and **4** under the same conditions (Table 4). The results clearly indicate that diiron-peroxo intermediates play a key role in the formation of the reactive species responsible for the H_2_O_2_ oxidation.

### 2.1. Kinetic Studies on the Formation of Diiron-Peroxo Complexes *(****5***–***8****)*

**Figure 3:**
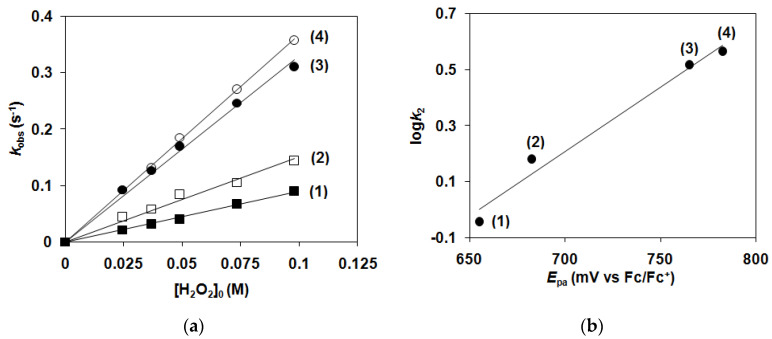
(**a**) Formation of diiron-peroxo (**5**–**8**) complexes. Plots of *k*_obs_ against H_2_O_2_ concentration to determine a second-order rate constant. [**1**–**4**]_0_ = 2 mM, in CH_3_CN at 15 °C. (**b**) Dependence of the formation rate of peroxo-diiron complexes (**5**–**8**) on the oxidation potential (*E*_pa_) of the [Fe^II^(L_1–4_)(solvent)_3_](ClO_4_)_2_ (**1**–**4**) complexes.

Detailed kinetic studies on the reaction of **1** (2 mM) with H_2_O_2_ (24–98 mM) were carried out in CH_3_CN at 15 °C, and the formation of the green species **5** was followed by UV-vis spectroscopy as an increase in absorbance at 690 nm under pseudo-first-order conditions (excess of H_2_O_2_) (Table 2). At constant [**1**]_0_ = 2 mM, the formation of **5** exhibits a first-order dependence, and the first-order rate constants show a good linear dependence with [H_2_O_2_]_0_ (Figure 3a), affording the second-order rate constant *k*_2_ = 0.93 M^−1^ s^−1^ at 15 °C and 1.09 M^−1^ s^−1^ at 20 °C. These values are about 5–6 times smaller than those obtained for [Fe^II^(MeBzim-Py)]^2+^ (5.5 M^−1^ s^−1^), Fe^II^(MeBzim-Py)^2+^ (6.6 M^−1^ s^−1^) and Fe^II^(TBA)^2+^ (6.54 M^−1^ s^−1^) complexes under the same conditions at 20 °C [23,26]. It is worth noting that adding large volumes of water (~0.5 M) resulted in nearly twice the rate of the diiron-peroxo formation. Furthermore, a kinetic isotope effect (KIE) of 2.99 and 3.07 were observed for the formation of **6** and **7**, when the experiments were carried out in the presence of added H_2_O or D_2_O. This value may represent the result of much more multiple effects compared to the elemental step, but clearly indicate the key role of the water during the diiron-peroxo complex formation.

**Figure 4:**
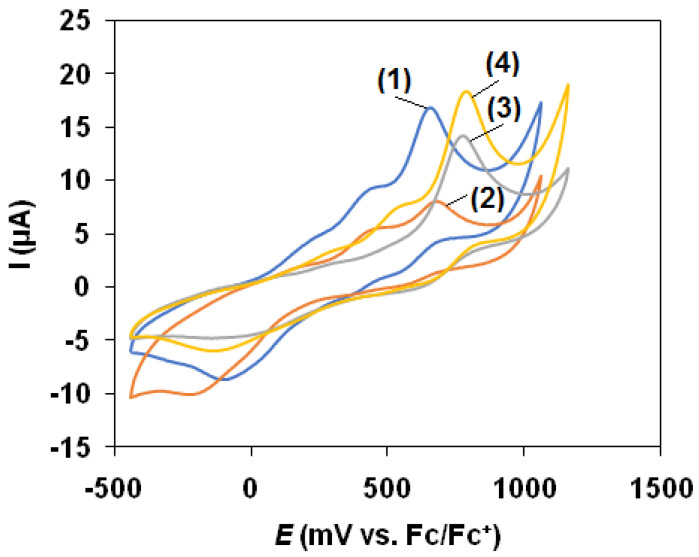
Cyclic voltammograms of [Fe^II^(L_1–4_)(solvent)_3_](ClO_4_)_2_ complexes (**1**–**4**) (5 mM in acetonitrile, supporting electrolyte: tetrabutylammonium-perchlorate (0.1 M), scan rate: 100 mV/s).

In the next step, we investigated the effect of the the ligand modification by introducing electron withdrawing (Cl) or electron releasing (CH_3_) substituents on the pyridine arms in the fourth and fifth positions with emphasis on the redox potential and Lewis acidity of the precursor complex. Since the redox potential of the substituted complexes can be measured under the same conditions, it can be used as excellent reactivity descriptor. On the cyclic voltammogram of the Fe(II)-isoindoline complexes (**1**–**4**) irreversible reduction and oxidation peaks can be recognized within the range of −440 to +1160 mV (vs. Fc/Fc^+^). The huge peak separations (Δ*E* = *E*_pa_ − *E*_pc_, Table 4) indicates the irreversibility of the redox processes and suggests chemical reactions coupled to the electrode reaction. The results, obtained at the scan rate of 100 mV/s, show that oxidation potential is mainly influenced by the electronic properties of the ligands, since the anodic peak potentials (*E*_pa_) of the complexes are increasing in the order of electron releasing ability of the substituent on the pyridine moiety (Figure 4). The *E*_pa_ spans a 128 mV range from 655 mV (**1**) to 783 mV (**4**), which is not too large but informative, in terms of the effect of the substituents. However, the fact that the anodic peak of complex **1** can be found at lower potentials than it could be expected according to electronic effects, suggest that steric effects are also influencing the oxidation reactions. One can assume that the steric effect of the methylated and chlorinated ligands can cause an anodic shift in the oxidation peak potential of the complexes. 

The four iron complexes, **1**–**4** have been compared to investigate the effect of the various aryl substituents. Figure 3a shows the results of the activity for the precursor complexes (**1**–**4**) during the formation of their appropriate diiron-peroxo intermediates (**5**–**8**). This demonstrates only small differences exist, based on the first-order rate constants obtained under the same conditions. It was found that complex **4** with 5-chloropyridyl side chains is the most reactive complex with *k*_obs_ of 3.66 M^−1^ s^−1^, whilst complex **1** with unsubstituted side chains was proved to be the least active with *k*_obs_ of 0.93 M^−1^ s^−1^ at 15 °C. Based on the CV data, we have got a clear evidence that the formation rate of the diiron-peroxo species (*k*_2_) increases almost linearly with the redox potential (*E*_pa_ for Fe^III^/Fe^II^) of the precursor complex (Figure 3b). In summary, the higher the redox potentials of Fe^III^/Fe^II^ redox couple the higher is the activity of the precursor complex towards to H_2_O_2_. Complexes with 4- and 5-chloro-pyridine side chains (**3** and **4**), whose metal sites are electron-deficient are faster in their reaction with H_2_O_2_ than electron-rich derivatives **1** and **2** with more Lewis basic side chains.

### 2.2. Catalase-Like Activity of Peroxo-Diiron Complexes

**Figure 5:**
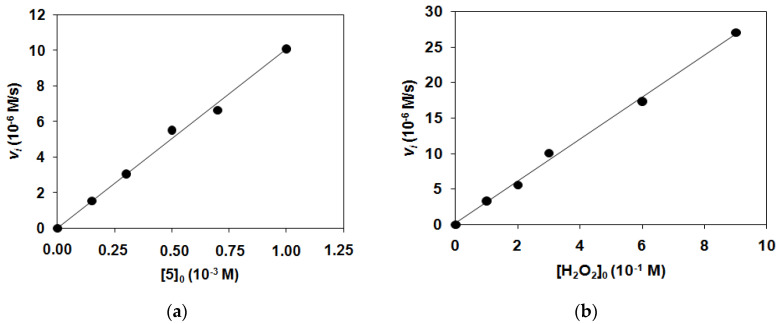
**5**-mediated disproportionation of H_2_O_2_ in CH_3_CN at 25 °C. (**a**) Dependence of the reaction rate (*V*_i_) for H_2_O_2_ oxidation on the diiron-peroxo complex **5** concentration. [H_2_O_2_]_0_ = 300 mM (**b**) Dependence of the reaction rate (*V*_i_) for H_2_O_2_ oxidation on the H_2_O_2_ concentration. [**5**]_0_ = 1 mM.

The ability of the in situ generated diiron-peroxo species (**5)** to mediate the disproportionation reaction of H_2_O_2_ into O_2_ and H_2_O was tested in CH_3_CN by UV-vis spectroscopy as a decrease in absorbance at 690 nm under pseudo-first-order conditions (excess of H_2_O_2_) (Table 3). The initial rate of decay of diiron-peroxo species was measured as a function of the complex and substrate concentrations in CH_3_CN. At constant [H_2_O_2_]_0_ = 300 mM, the disproportionation reaction shows first-order kinetic behavior on [5], and the first-order rate constant k_1_ = 4.968 × 10^−3^ s^−1^ was obtained at 20 °C from the slope of the plot of V_i_ versus [**5**]_0_ (Figure 5a). At constant [**5**]_0_ = 2 mM, initial rates (V_i_) exhibit a linear dependence with [H_2_O_2_]_0_ (Figure 5b) affording first-order rate constant k_1′_ = 29.63 × 10^−3^ s^−1^. The second-order rate constant, k_2_ = 3.4 M^−1^ s^−1^ was obtained from either k_1_/[H_2_O_2_]_0_ or k_1′_/[**5**]_0_. This value is much smaller than that was found for the analogue [Mn(L_1_)]^2+^ complex (k_2_(k_cat_/K_M_) = 79 ± 4 M^−1^ s^−1^) in protic solution, where Mn^IV^(O) intermediate was proposed as reactive species [29]. A kinetic isotope effect (KIE) of 1.73 and 1.66 were observed for the decay of **6** and **7**, when the experiments were carried out in the presence of added H_2_O or D_2_O. These results indicate the key role of the water during the diiron-peroxo mediated disproportionation reaction.

**Figure 6:**
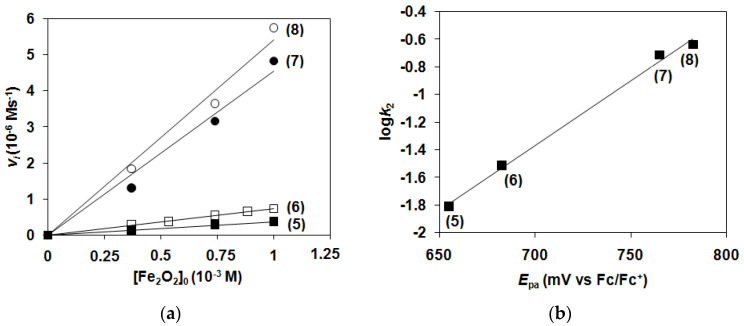
Peroxo-diiron complexes-mediated disproportionation of H_2_O_2_ in CH_3_CN at 15 °C. (**a**) Dependence of the reaction rate (*V*_i_) for H_2_O_2_ oxidation on the diiron-peroxo complex **5**–**8** concentration. [H_2_O_2_]_0_ = 24 mM (**b**) Dependence of the decay rate of peroxo-diiron complexes (**5**–**8**) on the oxidation potential (*E*_pa_) of the [Fe^II^(L_1–4_)(solvent)_3_](ClO_4_)_2_ (**1**–**4**) complexes.

In the next step, we investigated the effect of the ligand modification by introducing electron donating (-CH_3_) and electron withdrawing (-Cl) phenyl-ring substituents. The substituted ligand-containing diiron-peroxo intermediates **5**, **6**, **7** and **8** show an increasing rate in the listed order (Figure 6a). It was found that complex **8** with 5-chloropyridine side chains is the most efficient oxidant with the fastest rate *k*_2_ = 23.8 × 10^−2^ M^−1^ s^−1^, while complexes **5** and **6** with unsubstituted and 4-methylpyridine side chains are the less efficient oxidants with *k*_2_ = 1.52 × 10^−2^ M^−1^ s^−1^ and *k*_2_ = 3.05 × 10^−2^ M^−1^ s^−1^, respectively. These results give clear evidence that substituents affect the redox potential and the catalase activity of the complexes. Table 4 and Figure 6b show that the redox potentials (*E*_pa_) of the complexes and their activity increase with increasing electron-withdrawing ability of the substituent.

**Figure 7:**
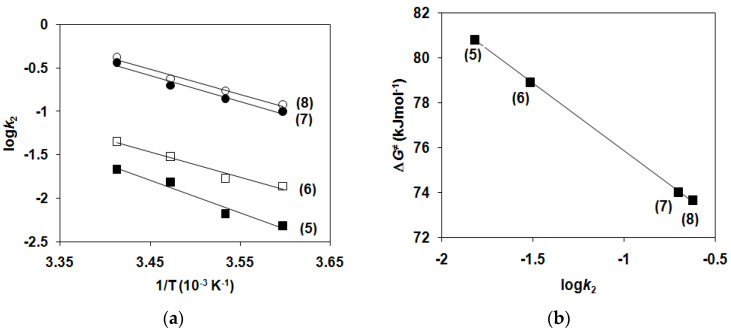
(**a**) Arrhenius plots of log*k*_2_ versus 1/T for the Fe^III^_2_(μ-O)(μ-1,2-O_2_)(L_1–4_)_2_(Solv)_2_]^2+^ (**5**–**8**)-mediated disproportionation of H_2_O_2_ in CH_3_CN. [**5**–**8**]_0_ = 2 mM, [H_2_O_2_]_0_ = 24 mM. (**b**) Plot of Δ*G*^≠^ versus lg*k*_2_ for the reaction of [Fe^III^_2_(μ-O)(μ-1,2-O_2_)(L_1–4_)_2_(Solv)_2_]^2+^ (**5**–**8**) with H_2_O_2_.

**Figure 8:**
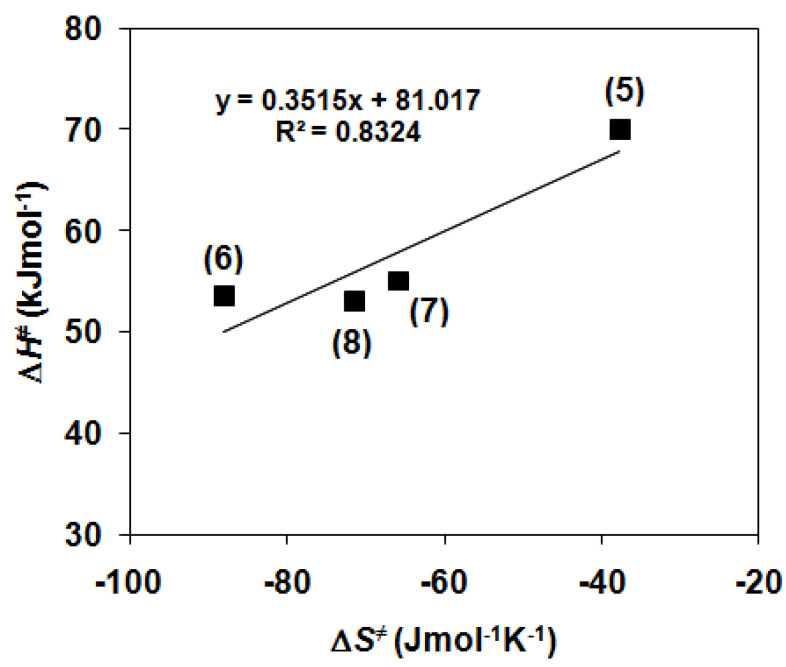
Isokinetic plot for the for the Fe^III^_2_(μ-O)(μ-1,2-O_2_)(L_1–4_)_2_(Solv)_2_]^2+^ (**5**–**8**)-mediated disproportionation of H_2_O_2_ in CH_3_CN.

Based on the temperature dependence of the reactivity of diiron-peroxo complexes (Figure 7a), the experimentally determined the difference between Δ*G*^≠^ values was 7.3 kJ mol^−1^. Significantly lower free activation energy value was obtained for the **8**-mediated disproportionation of H_2_O_2_ in comparison to **5**. The calculated Gibbs energy values correlate very well with the reaction rates (Figure 7b). The values of −TΔ*S*^≠^ were lower than ΔH^≠^ in the investigated temperature range, indicating an enthalpy-controlled reactions. As a result of a compensation effect increasing activation enthalpies are offset by increasingly poitive entropies yielding Δ*H*^≠^ = 81 kJ mol^−1^ at the intercept (Figure 8), which value is a little bit higher than that was obtained for the conversion alkylperoxo-iron(III) intermediates to oxoiron(IV) through O-O bond homolysis (Δ*H*^≠^ = 61.3 kJ mol^−1^) [30].

## 3. Experimental

### 3.1. Materials and Methods

The ligands 1,3-bis(2′-pyridylimino)isoindolines (L_1_ = indH, L_2_ = 4Me-indH, L_3_ = 4Cl-indH, L_4_ = 5Cl-indH,) and their complexes (**1**–**4**) were prepared according to published procedures [31]. All manipulations were performed under a pure argon atmosphere using standard Schlenk-type inert-gas techniques. Solvents used for the reactions were purified by literature methods and stored under argon. The starting materials for the ligand are commercially available and they were purchased from Sigma Aldrich. The UV-visible spectra were recorded on an Agilent 8453 diode-array spectrophotometer using quartz cells. IR spectra were recorded using a Thermo Nicolet Avatar 330 FT-IR instrument (Thermo Nicolet Corporation, Madison, WI, USA), Samples were prepared in the form of KBr pellets. Microanalyses elemental analysis was done by the Microanalytical Service of the University of Pannonia.

### 3.2. Characterization of Ligands and Their Complexes

indH: FT-IR (ATR) ν = 3199 (w), 3061 (w), 1622 (s), 1606 (m), 1577 (s), 1550 (s), 1454 (s), 1427 (s), 1373 (m), 1306 (m), 1259 (s), 1217 (s), 1139 (m), 1097 (m), 1042 (m), 991 (w), 885 (w), 875 (w), 859 (w), 839 (w), 808 (m), 792 (s), 783 (s), 769 (s), 737 (s), 708 (m), 696 (s), 689 (s), 627 (w). UV/Vis (DMF): λ_max_ (logε) = 317 (3.09), 332 (3.18), 348 (3.21), 368 (3.28), 386 (3.34), 410 (3.11) nm.

4Me-ind: FT-IR (ATR) ν = 3209 (w), 3049 (w), 1645 (m), 1627 (s), 1591 (s), 1541 (m), 1460 (s), 1408 (w), 1364 (m), 1305 (m), 1281 (m), 1242 (s), 1186 (m), 1132 (w), 1114 (w), 1101 (m), 1036 (m), 997 (w), 929 (m), 891 (m), 848 (w), 810 (s), 779 (m), 742 (w), 694 (s), 660 (w). UV/Vis (DMF): λ_max_ (logε) = 316 (2.99), 332 (3.08), 348 (3.12), 366 (3.18), 387 (3.21), 411 (2.98) nm.

4Cl-ind: FT-IR (ATR) ν = 3213 (w), 1743 (w), 1637 (m), 1624 (m), 1565 (s), 1539 (m), 1448 (s), 1359 (m), 1309 (m), 1300 (m), 1281 (w), 1219 (m), 1184 (w), 1089 (m), 1040 (m), 989 (m), 895 (s), 877 (s), 804 (m), 779 (m), 735 (s), 701 (s) 687 (m), 656 (w). UV/Vis (DMF): λ_max_ (logε) = 315 (3.16), 332 (3.27), 348 (3.29), 367 (3.31), 385 (3.35), 409 (3.12) nm.

5Cl-ind: FT-IR (ATR) ν = 3317 (w), 3228 (w), 1745 (m), 1633 (s), 1572 (s), 1470 (m), 1447 (s), 1359 (m), 1311 (m), 1256 (w), 1244 (w), 1228 (m), 1217 (m), 1184 (w), 1105 (s), 1034 (s), 1007 (m), 955 (w), 924 (w), 880 (w), 843 (s), 773 (s), 754 (m), 725 (w), 702 (s), 691 (s), 638 (m), 619 (w), 602 (m). UV/Vis (DMF): λ_max_ (logε) = 338 (3.15), 354 (3.15), 376 (3.19), 395 (3.25), 421 (3.02) nm.

**1**: FT-IR (ATR) ν = 3329 (w), 1728 (w), 1654 (m), 1626 (s), 1614 (m), 1593 (w), 1554 (w), 1524 (s), 1485 (s), 1467 (m), 1433 (m), 1373 (w), 1304 (w), 1262 (w), 1207 (m), 1055 (s), 929 (w), 860 (w), 779 (s), 714 (m), 621 (m). C_24_H_20_Cl_2_FeN_8_O_8_ (675.22): calcd. C 42.69, H 2.99, Cl 10.15, N 16.60; found C 42.82, H 2.93, Cl 10.11, N 16.42. UV/Vis (CH_3_CN): λ_max_ (logε) = 315 (3.36), 329 (3.45), 345 (3.48), 365 (3.56), 382 (3.61), 406 (3.37) nm.

**2**: FT-IR (ATR) ν = 3456 (w), 1734 (w), 1665 (m), 1654 (s), 1605 (s), 1551 (m), 1520 (s), 1493 (s), 1477 (m), 1367 (w), 1313 (w), 1279 (m), 1244 (w), 1215 (w), 1049 (s), 941 (w), 827 (w), 777 (w), 752 (w), 710 (m), 621 (m). C_26_H_27_Cl_2_FeN_8_O_8_ (706.29): calcd. C 44.21, H 3.85, Cl 10.04, N 15.87; found C 43.99, H 3.64, Cl 9.58, N 15.79. UV/Vis (CH_3_CN): λ_max_ (logε) = 315 (3.48), 328 (3.58), 345 (3.62), 365 (3.71), 383 (3.76), 406 (3.50) nm.

**3:** FT-IR (ATR) ν = 3366 (w), 3217 (w), 1773 (w), 1724 (w), 1670(m), 1628 (m), 1564 (m), 1543 (m), 1516 (s), 1483 (m), 1460 (s), 1362 (w), 1306 (w), 1228 (w), 1205 (m), 1090 (s), 1051 (s), 910 (m), 867 (w), 823 (w), 744 (s), 710 (s), 621 (m). C_24_H_21_Cl_4_FeN_8_O_8_ (747.13): calcd. C 38.58, H 2.83, Cl 18.98, N 15.00; found C 38.25, H 2.95, Cl 18.63, N 15.52. UV/Vis (CH_3_CN): λ_max_ (logε) = 328 (3.10), 344 (3.09), 363 (3.04), 383 (2.99), 406 (2.71) nm.

**4:** FT-IR (ATR) ν = 3323 (w), 3228 (w), 1773 (w), 1717 (w), 1651(w), 1628 (m), 1612 (m), 1546 (m), 1521 (s), 1477 (s), 1458 (s), 1391 (w), 1366 (m), 1335 (w), 1298 (w), 1261 (w), 1236 (m), 1205 (m), 1096 (s), 1076 (s), 922 (m), 851 (w), 835 (m), 781 (w), 752 (m), 711 (s), 650 (w), 621 (m). C_24_H_21_Cl_4_FeN_8_O_8_ (747.13): calcd. C 38.58, H 2.83, Cl 18.98, N 15.00; found C 38.81, H 3.04, Cl 18.63, N 15.39. UV/Vis (CH_3_CN): λ_max_ (logε) = 335 (2.64), 353 (2.59), 373 (2.47), 395 (2.36), 418 (2.04) nm.

### 3.3. Cyclic Voltammetry

Cyclic voltammetry experiments were performed on a Radiometer Analytical PGZ-301 potentiostat with a conventional three-electrode configuration, consisting of a glassy carbon working electrode (ID = 3 mm), a platinum wire auxiliary electrode, and Ag/AgCl reference electrode. Potentials are referenced to the Fc/Fc^+^ redox couple. Ferrocene was added as an internal standard at the end of the experiments. Procedure for the cyclic voltammetry experiment: 0.05 mmol of **1**–**4** was dissolved in 10 mL dry acetonitrile, containing 349.1 mg (1 mmol) of tetrabutylammonium-perchlorate, which served as supporting electrolyte. The solution was bubbled with argon to remove dissolved gas residuals and to ensure inert atmosphere during measurements. The working electrode was wet polished on 0.5 μm alumina slurry or emery paper grade 500, after each measurement. Cyclic voltammograms were recorded with a scan rate of 100 mV s^−1^.

### 3.4. Generation of Diiron-Peroxo Complexes *(****5***–***8****)*

Precursor complexes (**1**–**4**) were dissolved in 1.5 mL acetonitrile and added 4 equivalent H_2_O_2_, in different temperature (0, 5, 10 15 °C) and the reactions were followed with UV-Vis spectroscopy at 680 nm, the cuvette length was 1 cm.

### 3.5. Diiron-Peroxo-Mediated Disproportionaton of H_2_O_2_

Reactions were carried out by mixing the substrate (H_2_O_2_) with the in situ generated complex **5**–**8** (1 mM) in 1.5 mL acetonitrile in different temperature (0, 5, 10 15 °C) and the reactions were followed with UV-Vis spectroscopy at 680 nm, the cuvette length was 1 cm. To ensure the reliability of the measurements, 2–3 parallel measurements were performed.

Catalytic reactions were carried out at 20 °C in a 30 cm^3^ reactor containing stirring bar under air. In a typical experiment the complex was dissolved dissolved in 20 cm^3^ CH_3_CN, and the flask was closed with a rubber septum. H_2_O_2_ was injected by syringe through the septum. The reactor was connected to a graduated burette filled with oil, and the evolved dioxygen was measured volumetrically at time intervals of 2 s.

## 4. Conclusions

**Scheme 3:**
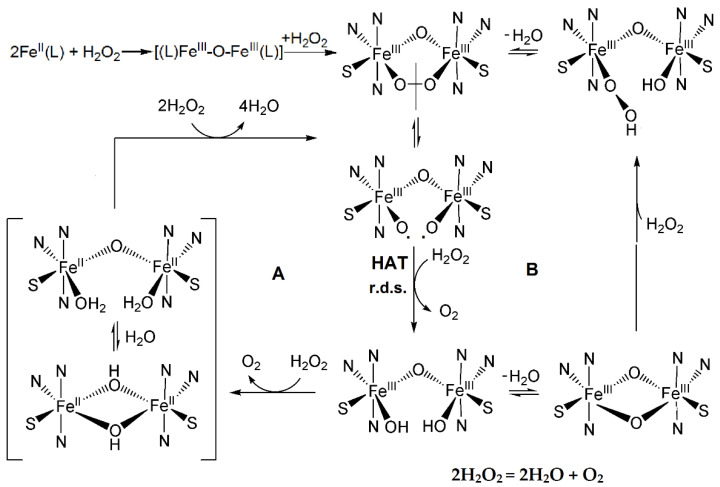
Proposed mechanism for the formation of Fe^III^_2_(μ-O)(μ-1,2-O_2_)(L_1_-L_4_)_2_(Solv)_2_]^2+^ (**5**–**8**) complexes and their catalase-like reactions.

In summary, the results of the reaction kinetics and CV measurements are shown in Table 4. Based on these data the following reaction mechanism, including the diiron-peroxo complex formation, and its reaction with H_2_O_2_, was proposed (Scheme 3). We obtained clear evidence for the role of the peroxo-intermediate in diiron catalase mimics. We also found that the higher the redox potentials of the Fe^III^/Fe^II^ redox couple, the higher the catalase-like activity, and the complexes with electron-deficient metal sites are significantly more reactive. It can be explained by their electrophilic character. The results obtained may contribute to the elucidation of the mechanism of both dimanganase and diiron-containing catalase enzymes.

## Figures and Tables

**Table 1:** Dependence of the yield of peroxo complexes (**5**–**8**) and the evolved oxygen (parantheses) on the number of cycles. [Fe]_0_ = 2 mM, [H_2_O_2_]_0_ = 24 mM (4×) in CH_3_CN at 20 °C.

Cycles	Yield (5) and (O_2_)/%	Yield (6)/%	Yield (7)/%	Yield (8)/%
I.	100 (95)	100	100	100
II.	91 (92)	87	48	43
III.	73 (78)	70	39	20
IV.	61 (64)	53	31	13
V.	41 (27)	36	26	12

**Table 2:** Kinetic data for the formation of intermediates **5**, **6**, **7** and **8** in CH_3_CN.

Nr.	Complex	T/°C	[1–4]_0_/mM	[H_2_O_2_]_0_/M	*k*_obs_10^−2^ s^−1^	*k*_2_/M^−1^ s^−1^
1	**1**	15	2	0.0243	2.12	0.87 ± 0.03
2	**1**	15	2	0.0367	3.15	0.86 ± 0.04
3	**1**	15	2	0.0489	4.07	0.83 ± 0.03
4	**1**	15	2	0.0734	6.76	0.92 ± 0.04
5	**1**	15	2	0.0979	9.04	0.92 ± 0.03
6	**2**	15	2	0.0243	4.45	1.83 ± 0.09
7	**2**	15	2	0.0367	5.83	1.58 ± 0.05
8	**2**	15	2	0.0489	8.44	1.72 ± 0.06
9	**2**	15	2	0.0734	10.5	1.43 ± 0.04
10	**2**	15	2	0.0979	14.45	1.48 ± 0.04
11	**3**	15	2	0.0243	9.22	3.79 ± 0.15
12	**3**	15	2	0.0367	12.64	3.44 ± 0.13
13	**3**	15	2	0.0489	16.95	3.46 ± 0.14
14	**3**	15	2	0.0734	24.61	3.35 ± 0.14
15	**3**	15	2	0.0979	31.07	3.17 ± 0.11
16	**4**	15	2	0.0243	9.25	3.81 ± 0.15
17	**4**	15	2	0.0367	13.17	3.59 ± 0.14
18	**4**	15	2	0.0489	18.48	3.78 ± 0.16
19	**4**	15	2	0.0734	27.07	3.69 ± 0.15
20	**4**	15	2	0.0979	35.83	3.66 ± 0.15

**Table 3:** Kinetic data for the disproportionation of H_2_O_2_ mediated by **5**, **6**, **7** and **8** in CH_3_CN.

Nr.	Complex	T/°C	[1–4]_0_/mM	[H_2_O_2_]_0_/M	*k*_obs_/10^−3^ s^−1^	*k*_2_/10^−2^ M^−1^ s^−1^
1	**1**	15	0.738	0.0243	0.358	1.47 ± 0.06
2	**1**	15	1.48	0.0243	0.387	1.59 ± 0.07
3	**1**	15	2	0.0243	0.370	1.52 ± 0.06
4	**1**	5	2	0.0243	0.116	0.477 ± 0.02
5	**1**	10	2	0.0243	0.160	0.658 ± 0.03
6	**1**	20	2	0.0243	0.523	2.15 ± 0.11
7	**1**	25	2	0.1	3.33	3.33 ± 0.11
8	**1**	25	2	0.2	5.57	2.79 ± 0.08
9	**1**	25	2	0.3	10.1	3.36 ± 0.09
10	**1**	25	2	0.6	17.3	2.89 ± 0.07
11	**1**	25	2	0.9	27.1	3.01 ± 0.06
12	**1**	25	0.3	0.3	10.3	3.42 ± 0.12
13	**1**	25	0.6	0.3	10.1	3.38 ± 0.11
14	**1**	25	1	0.3	11.1	3.68 ± 0.14
15	**1**	25	1.4	0.3	9.47	3.16 ± 0.13
16	**1**	10	2	0.3	1.99	0.663 ± 0.02
17	**1**	15	2	0.3	3.63	1.21 ± 0.05
18	**1**	20	2	0.3	6.12	2.04 ± 0.08
19	**2**	15	0.738	0.0243	0.823	3.39 ± 0.11
20	**2**	15	1.07	0.0243	0.692	2.85 ± 0.09
21	**2**	15	1.48	0.0243	0.772	3.18 ± 0.10
22	**2**	15	1.77	0.0243	0.744	3.06 ± 0.09
23	**2**	15	2	0.0243	0.730	3.00 ± 0.11
24	**2**	5	2	0.0243	0.332	1.37 ± 0.04
25	**2**	10	2	0.0243	0.413	1.70 ± 0.05
26	**2**	20	2	0.0243	1.09	4.49 ± 0.27
27	**3**	15	0.738	0.0243	3.92	16.1 ± 0.72
28	**3**	15	1.48	0.0243	4.71	19.4 ± 0.62
29	**3**	15	2	0.0243	4.83	19.9 ± 0.68
30	**3**	5	2	0.0243	2.42	9.96 ± 0.35
31	**3**	10	2	0.0243	3.41	14.01 ± 0.63
32	**3**	20	2	0.0243	8.90	36.6 ± 2.01
33	**4**	15	0.738	0.0243	5.49	22.6 ± 0.79
34	**4**	15	1.48	0.0243	5.44	22.4 ± 0.83
35	**4**	15	2	0.0243	5.75	23.8 ± 0.85
36	**4**	5	2	0.0243	2.89	11.9 ± 0.48
37	**4**	10	2	0.0243	4.20	17.3 ± 0.67
38	**4**	20	2	0.0243	10.2	42.01 ± 1.77

**Table 4:** Rate constants, electrochemical data and activation parameters for the formation of Fe^III^_2_(μ-O)(μ-1,2-O_2_)(L_1_-L_4_)_2_(Solv)_2_]^2+^ (**5–8**) complexes and their catalase-like reactions.

	1 (5)	2 (6)	3 (7)	4 (8)
*E*_pa_(Fe^II^/Fe^III^)/mV vs. Fc/Fc^+^	655	683	765	783
*E*_pc_(Fe^II^/Fe^III^)/(mV vs. Fc/Fc^+^	−90	−223	−155	−143
Δ*E*/mV	745	905	920	925
yield/%	85	100	92	85
TON ([S]/[Fe])	10.4	12.2	11.3	10.4
TOF ([S]/[Fe]/h)	288	418	354	234
*k*_2_(cat)/10^−2^ M^−1^ s^−1^ (15 °C)	1.52	3.05	19.9	23.8
*E*_A/_kJ mol^−1^	72.3	55.9	57.5	55.5
Δ*S*^≠^/J mol^−1^ K^−1^	−37.7	−88.1	−65.9	−71.4
Δ*H*^≠/^kJ mol^−1^	70	53.5	55	53.1
Δ*G*^≠^/kJ mol^−1^	81	78.9	74	73.7
*k*_decay_/10^−2^s^−1^ (15 °C)	0.13	0.15	1.22	1.41
*k*_form_/M^−1^ s^−1^ (15 °C)	0.93	1.43	3.16	3.66

## Data Availability

Not available.

## Sample Availability

Not available.
